# Identification of microRNA editing sites in three subtypes of leukemia

**DOI:** 10.3389/fmolb.2022.1014288

**Published:** 2022-11-14

**Authors:** Wenping Xie, Jun Yang, Nan Zhou, Hao Ding, Guangchen Zhou, Shuai Wu, Shiyong Guo, Wanran Li, Lei Zhang, Huaide Yang, Chunyi Mao, Yun Zheng

**Affiliations:** ^1^ Faculty of Life Science and Technology, Kunming University of Science and Technology, Kunming, Yunnan, China; ^2^ State Key Laboratory of Primate Biomedical Research, Institute of Primate Translational Medicine, Kunming University of Science and Technology, Kunming, Yunnan, China; ^3^ Yunnan Police College, Kunming, Yunnan, China; ^4^ Faculty of Information Engineering and Automation, Kunming University of Science and Technology, Kunming, Yunnan, China; ^5^ College of Horticulture and Landscape, Yunnan Agricultural University, Kunming, Yunnan, China

**Keywords:** leukemia, miRNA, miRNA editing, hsa-mir-21-5p, hsa-mir-155-5p, hsa-let-7b-5p

## Abstract

Leukemia is an aberrant hyper-proliferation of immature blood cells that do not form solid tumors. The transcriptomes of microRNAs (miRNAs) of leukemia have been intensively explored. However, miRNA editing of leukemia has not been extensively studied. To identify miRNA editing patterns and explore their functional relevance in leukemia, we analyzed 200 small RNA sequencing profiles of three subtypes of leukemia and identified hundreds of miRNA editing sites in three subtypes of leukemia. Then, we compared the editing levels of identified miRNA editing sites in leukemia and normal controls. Many miRNAs were differential edited in different subtypes of leukemia. We also found the editing levels of 3′-A editing sites of hsa-mir-21-5p and hsa-mir-155-5p decreased in chronic lymphocytic leukemia patients with radiation treatments. By integrating PAR-CLIP sequencing profiles, we predicted the targets of original and edited miRNAs. One of the edited miRNA, hsa-let-7b_5c, with an additional cytosine at 5′ end of hsa-let-7b-5p, potentially targeted VBP1 and CTDSP1. CTDSP1 was significantly downregulated in T-ALL compared to normal controls, which might be originated from the hyperediting of hsa-let-7b-5p in T-ALL. Our study provides a comprehensive view of miRNA editing in three different subtypes of leukemia.

## 1 Introduction

Leukemia is the most common malignant tumor originating from the hematopoietic system (Konopleva and Han, 2017). Leukemia is a highly heterogeneous malignant clonal disease manifested by uncontrolled proliferation of hematopoietic cells, which seriously affects bone marrow and lymphatic system, leading to high mortality (Gong et al., 2017; Konopleva and Han, 2017). Leukemia can mainly be classified according to its mode of progression (acute or chronic) and the affected lineage (lymphoid or myeloid). The four main subtypes are acute lymphocytic leukemia (ALL), chronic lymphocytic leukemia (CLL), acute myeloid leukemia (AML) and chronic myelogenous leukemia (CML) (Nemkov et al., 2019). ALL is the most common malignant disease in children (Onciu, 2009). According to the source of lymphocytes, it can be further divided into B lymphocyte ALL (B-ALL) and T lymphocyte ALL (T-ALL) (Rodriguez et al., 2021). Compared with B-ALL, T-ALL has an increased risk of treatment failure and recurrence (Onciu, 2009). CLL is a heterogeneous lymphocytic proliferative disease and is the most common form of leukemia in western countries, usually occurs in elderly patients, with a median age of diagnosis of 72 years (Iwona and Jacek, 2015; Qian et al., 2021). AML is an aggressive malignancy characterized by rapid proliferation of immature myeloid leukemia cells (Doehner et al., 2013). In most cases, this disease involves a bone marrow malignancy, although malignant cells may also be detected in peripheral blood or as extramedullary infiltration (Doehner et al., 2013; Nepstad et al., 2020). CML is a myeloproliferative neoplasm characterized by the Philadelphia chromosome (Ph) that affects one to two per 100,000 new patients per year and comprises 15% of leukemias in adults (How et al., 2021). The disease is driven by a reciprocal translocation of chromosomes 9 and 22, which results in the BCR-ABL fusion protein and dysregulated tyrosine kinase activity (Shallis et al., 2020).

In addition, there are other rarer types of leukemia, such as PLL (Cross and Dearden, 2019) and ATLL (Shah et al., 2018). Prolymphocytic leukemias (PLL) are mature lymphoid malignancies of either B-cell (B-PLL) or T-cell (T-PLL) lineage (Cross and Dearden, 2019). They are rare, accounting for approximately 3% of all mature lymphoid leukemias (Cross and Dearden, 2019). T cell prolymphocytic leukemia (T-PLL) is a mature T cell leukemia of small to medium size lymphocytes that shows post-thymic features (Ramos et al., 2021). It involves the peripheral blood, lymph nodes, spleen, liver, skin, pleura, central nervous system (CNS), and bone marrow (Ramos et al., 2021). Currently, more and more evidences indicate that chromosomal abnormalities play a role in the pathogenesis of T-PLL (Shi et al., 2018).

MiRNAs are highly conserved short non-coding RNAs with 22 nucleotide which bind and post-transcriptionally regulate mRNA (Simonson and Das, 2015). miRNAs play a variety of important regulatory roles in cells, including participating in the regulation of cell proliferation, differentiation, aging and death, inflammatory reaction, tumogenesis and other physiological and pathological processes, which is of great significance for the study of diseases, and their abnormal expression contributes to cell transformation and tumogenesis (Syeda et al., 2020). Recent studies have shown that miRNAs play an important role in the regulation of multiple leukemia subtypes, and they form a network of post-transcriptional regulation of gene expression in leukemia (Correia and Barata, 2020; Su et al., 2020; Yun et al., 2020). T-ALL is the result of a multi-step carcinogenic process (Fang, 2018). Many biologically related genetic and epigenetic changes have been identified as drivers of this process (Fang, 2018). Recently, more deep sequencing studies have shown that the molecular genetic subtypes of human T-ALL also exhibit unique miRNA expression characteristics (Wallaert et al., 2017). And many miRNAs have been identified by studying mutations in non-coding regions of the genome that lead to abnormal expression of transcription factors in T-ALL (Qian et al., 2016). For example, the tumor suppressive activity in T-ALL is at least partially attributable to ARRB1-mediated degradation of NOTCH1 (Shu et al., 2019). miR-223 targeted the 3 ’-UTR of ARRB1 to inhibit ARRB1 expression and effectively antagonized the tumor suppressive effect of ARRB1 in T-ALL (Shu et al., 2019). In addition, more and more miRNAs have been shown to have prognostic value in CLL. miR-34a targeted ZAP70 mRNA which is associated with chemotherapeutic refractory diseases (Balatti et al., 2015). Overexpression of miR-155, miR-29a, and miR-29b leads to the development of invasive CLL (Balatti et al., 2020). Among the most frequently occurring genetic events, deletion of chromosome 13q14 (del13q14) is considered to be one of the early causes of CLL and is the most common genetic abnormality of this leukemia, which is present in more than 50% of CLL cases (Calin et al., 2002). Del13q14 leads to the loss of two miRNAs, namely miR-15a and miR-16 (Calin et al., 2002). Therefore, the loss of miR-15a and miR-16 will eliminate or reduce the negative control for BCL2 translation, leading to the improvement of BCL2 protein expression level, thus BCL2 can contribute to the survival of CLL cells (Cimmino et al., 2005). At present, regulatory networks based on the profile of miRNA have few been described for T-PLL. Braun et al. found that more activated T cell phenotypes and more aggressive disease presentations were associated with increased expression of miR-223-3p as well as reduced expression of miR-21 and the miR-29 cluster (Braun et al., 2022).

RNA editing is a concerned mechanism in eukaryote and can alter the primary sequences of RNA transcripts by deleting, inserting and modifying residues (Nishikura, 2010). miRNAs are edited in several ways, including 3′ non-templated addition (3′-NTA) (Burroughs et al., 2010), Adenosine-to-Inosine (A-to-I) editing performed by adenosine deaminase (ADAR) and C-to-U (Cytidine-to-uridine) editing performed by apolipoprotein B mRNA editing polypeptide-like (APOBEC) enzymes (Galeano et al., 2012; Guo et al., 2022). Many studies have reported that changes in miRNA editing could both play a role in tumor pathogenesis and progression, and be used as early and specific biomarkers of selected forms of cancer (Guo et al., 2022). The A-to-I edited miR-376a inhibits glioma cell migration and invasion by targeting AMFR, and fails to inhibit the original target RAP2A (Choudhury et al., 2012). And the A-to-I edited miR-589-3p inhibits glioblastoma cell proliferation, migration and invasion by re-targeting the oncogene ADAM12 (Cesarini et al., 2018). Shoshan et al. (2015) showed that unedited miR-455-5p enhanced melanoma growth and metastasis *in vivo*, while the A-to-I edited miR-455-5p inhibited these features by upregulating tumor suppressor CPEB1. In addition, the A-to-I edited miR-378a prevents melanoma progression by regulating PARVA expression (Velazquez-Torres et al., 2018). Nigita et al. (2018) found that compared with normal tissues, the A-to-I edited miR-411-5p was significantly dysregulated in tissues and exosomes of patients with non-small cell lung cancer. Moreover, PAPD4 (PAP associated domain containing 4) stabilizes the miR-122 by 3′ adenylation, thereby stimulating translational repression of the cytoplasmic polyadenylation element-binding protein CPEB by miR-122 (Katoh et al., 2009). Wang et al. (2017) systematically characterized the miRNA editing profiles of 8,595 samples across 20 cancer types from miRNA sequencing Data of The Cancer Genome Atlas. A total of three confident A-to-I editing events were identified in at least 10% of 188 AML samples (Wang et al., 2017). Zipeto et al. (2016) showed targeting the RNA editing enzyme ADAR1 restores expression of let-7 and efficiently kills LSCs (leukemia stem cell), providing an innovative therapeutic target in CML. Recently, Gassner et al. (2020) reported that C-to-U and A-to-I editing occurred in 11 (3.5%) and 14 (4.4%) of the 315 miRNAs expressed, respectively, and these miRNA editing events contributed to deregulated mRNA network targeted by edited miRNAs.

However, other types of editing, such as 3′-nontemplated additions, of miRNAs were largely unknown in leukemia. To carefully examine the miRNA editing events and their potential functional roles in leukemia, we analyzed 200 small RNA sequencing profiles to characterize miRNA editing in T-ALL, CLL, and T-PLL. Our results provided a comprehensive view of different types of miRNA editing in three different leukemia subtypes.

## 2 Materials and methods

### 2.1 Small RNA sequencing profiles used

To comprehensively identify miRNA mutation and editing (M/E) sites in leukemia, we collected 200 small RNA sequencing profiles from the NCBI SRA database for three leukemia subtypes including T-cell acute lymphocytic leukemia (T-ALL), chronic lymphocytic leukemia (CLL), and T-cell prelymphocytic leukemia (T-PLL) (as listed in Supplementary Table S1). The qualities of the sRNA profiles were evaluated with the FastQC program (Andrews, 2010).

### 2.2 Genome and annotation of miRNAs

The unmasked human genomic sequences (hg38) were downloaded from UCSC Genome Browser (Rosenbloom et al., 2015), and the index files of human genome were generated with the bowtie-build program in the Bowtie package (Langmead et al., 2009) for subsequent analysis. The sequences of pre-miRNAs and miRNA annotation file in gff3 format were downloaded from the miRbase (release 21) (Kozomara and Griffiths-Jones, 2014).

### 2.3 Identifying miRNA mutation and editing sites in leukemia

The MiRME pipeline (Zheng et al., 2016) with the default settings was used to identify M/E sites in the selected sRNA-seq profiles. Briefly, the raw reads were examined to keep the qualified reads of which the sequencing scores of the first 25 nucleotides had sequencing scores of 30 or higher. Then, reads with at least 18 nt were kept after removing the 3′ adapters. The kept reads were aligned to pre-miRNAs using BLASTN with the options of ‘-S 1 -m 8 -e 0.01′ and the reads mapped to pre-miRNAs were retrieved. Then, these reads mapped to pre-miRNAs were aligned to the genome using Bowtie (v1.0.0) (Langmead et al., 2009) with the options of ‘-a -best -S -v 1’. The alignments of reads to genome were checked by the cross-mapping correction algorithm (de Hoon et al., 2010) to calculate the weights or percentages of reads at different genomic loci of reads. Then, the obtained results of different samples were combined by a separate program in the MiRME package (see details in (Zheng et al., 2016; Zheng, 2018)). Based on the positions of M/E sites in miRNAs and mutations in dbSNP, the identified M/E sites were classified into nine different editing types, i.e., A-to-I, C-to-U, 3′-A, 3′-U, 3′-Other, 5′-editing, Other, SNP and Pseudo (Zheng et al., 2016).

The following criteria were used to define M/E sites with significant editing levels: (i) the relative level of editing was at least 5%; (ii) at least 10 reads supported the editing event; (iii) the score threshold of sequencing reads was 30; and (iv) a multiple-test corrected *p*-value using the Benjamini and Hochberg method (Benjamini and Hochberg, 1995) of smaller than 0.05; (v) only M/E sites that had significant editing levels in 20% of all the samples were kept in further analysis. The predicted M/E sites that belonged to A-to-I, C-to-U and Other were manually examined.

To remove M/E sites due to random sequencing errors, 591 M/E sites that had significant editing levels in at least 20% (10 samples) of the T-ALL (n = 48) and T-ALLcontrol (n = 4) samples used in this study were kept in further analysis. 646 M/E sites that had significant editing levels in at least 20% (5 samples) of the CLLblood (n = 23) and CLLcontrol (n = 2) samples used in this study were kept. 381 M/E sites that had significant editing levels in at least 20% (11 samples) of the T-PLL (n = 21) and T-PLLcontrol (n = 35) samples used in this study were kept. And 394 M/E sites that had significant editing levels in at least 20% (14 samples) of the CLL-Ir (n = 35) and CLL-Un (n = 35) samples used in this study were kept in further analysis.

Afterwards, the identified M/E sites were compared to reported editing sites in miRNAs in the DARNED database (Kiran and Baranov, 2010), the RADAR database (Ramaswami and Li, 2014) and literature (Kawahara et al., 2008; Alon et al., 2012; Peng et al., 2012; Gong et al., 2014; Zheng et al., 2016; Guo et al., 2022).

The identified M/E site was named by the pre-miRNA name, the position of M/E site in pre-miRNA, original nucleotide in upper case, and edited/mutated nucleotide in lower case. For example, hsa-mir-376a-1_9_A_g means an A-to-I editing event at the ninth nucleotide of hsa-mir-376a-1, A is original nucleotide, and g is edited/mutated nucleotide. And edited miRNA was named by the pre-miRNA name, the position of M/E site in pre-miRNA, and the edited/mutated nucleotide in lower case. For example, hsa-mir-376a-1_9g is the A-to-I edited miR-397a-1-5p.

### 2.4 Comparing to reported SNPs in the M/E sites

The identified M/E sites were compared to reported SNPs in miRNAs reported previously (Han and Zheng, 2013) (which was based on the dbSNP v137) and compared to the reported SNPs in the dbSNP (v151) database. Only sites that satisfied the following criteria were regarded as SNPs, (i) had the same genomic positions as the SNPs, (ii) had the same nucleotides as the alleles of the SNPs for both the original and changed nucleotides, and (iii) had editing levels of 100% in at least one of the samples selected.

### 2.5 Clustering and principle component analysis using the editing levels of M/E sites

We performed PCA analysis using the editing levels of the editing sites in leukemia and their control samples. The princomp function in MATLAB were used in the clustering analysis. We normalized the expression levels of original miRNAs and edited miRNAs, and the editing level of the M/E sites with the quantilenorm function in MATLAB (as shown in Supplementary Table S11). Next, we used the normalized value plus one to calculating the correlation coefficient between samples, and one minus correlation coefficient values as the distances of between samples, and the ward D2 method was used for clustering analysis. Then the hclust function in R was used to perform hierarchical clustering.

### 2.6 Identifying miRNA M/E sites with different editing levels in leukemia

As shown in Supplementary Table S6, the editing levels of 591 M/E sites in T-ALL (n = 48) and T-ALLcontrol (n = 4) samples, the editing levels of 646 M/E sites in the CLLblood (n = 23) and CLLcontrol (n = 2) samples, the editing levels of 381 M/E sites in T-PLL (n = 21) and T-PLLcontrol (n = 35) samples, and the editing levels of 394 M/E sites in CLL-Ir (n = 35) and CLL-Un (n = 35) samples were compared with the Mann-Whitney *U*-tests respectively. The obtained *p*-values were corrected with the Benjamini–Hochberg correction method (Benjamini and Hochberg, 1995) respectively. M/E sites with corrected *p*-values smaller than 0.05 were regarded as having significantly different editing levels in leukemia samples compared to their control samples.

### 2.7 Identifying targets of original and edited miRNAs

We selected 5′-editing M/E sites whose mature miRNAs had an additional C at 5′ end with significantly different editing levels in T-ALL, T-PLL samples compared to their control samples respectively, to identify targets for original and edited miRNAs. The targets of original and edited miRNAs were predicted using the MiCPAR algorithm (Zheng, 2018) with its default parameters. As shown in Supplementary Tables S1.4, 11 PAR-CLIP sequencing profiles were downloaded from NCBI SRA database with series accession number SRP002487 (including seven from HEK293 cells stably expressing FLAG/HA -tagged AGO1, AGO2, AGO3, and AGO4 (Hafner et al., 2010) and SRP018015 (from HEK293 cell lines stably expressing HIS/FLAG/HA-tagged AGO1 and AGO2 (Memczak et al., 2013)). The raw reads from 11 PAR-CLIP sequencing files were then filtered and processed to obtain qualified reads. The remaining reads in these 11 profiles were combined and used in the identification of miRNA targets with the MiCPAR algorithm. The annotation of NCBI RefSeq genes in the GTF file, the mRNA sequences of NCBI RefSeq genes and soft-masked genome sequences of human (version hg38) were downloaded from the UCSC Genome Browser and used as inputs of the MiCPAR algorithm. The targets with at least 1 PAR-CLIP read with T-to-C variation were kept for further analysis.

The gene expression profiles of T-ALL patients and normal control (Yang L. et al., 2021) were used to examine the expression of miRNA targets. We compared 18 samples of T-ALL patients and four samples of normal control with edgeR. Gene with significantly different expression in T-ALL patients were defined as corrected *p*-value < 0.05.

### 2.8 GO and pathway analysis for the original and edited miRNAs

The GO (Gene Ontology) terms and KEGG (Kyoto Encyclopedia of Genes and Genomes) (Kanehisa and Goto, 2000; Kanehisa, 2019, 2021) pathway enrichment of the targets of the original or edited miRNAs were analyzed with KOBAS (v3) (Xie et al., 2011), respectively. The enriched GO terms were divided into three main categories, i.e., Biological Process, Cellular Component and Molecular Function. Then, the enriched GO terms and KEGG pathways of the original and edited miRNAs were compared. After comparing the targets of original and edited miRNAs, new targets of edited miRNAs were identified, and then KOBAS was used again for GO term and KEGG pathway enrichment analysis of the new targets of edited miRNAs.

## 3 Results

### 3.1 Summary of small RNA sequencing data used

As listed in Supplementary Table S1, we collected 200 small RNA sequencing profiles from the NCBI SRA database for three leukemia subtypes including T-cell acute lymphocytic leukemia (T-ALL), chronic lymphocytic leukemia (CLL), and T-cell prelymphocytic leukemia (T-PLL). These profiles included 48 pediatric T-ALL samples (36 blood and 12 bone marrow samples) and four normal thymocytes samples from healthy people (used as normal controls of T-ALL, briefly as T-ALLcontrol), 21 CLL whole blood samples (CLLblood) and two whole blood samples of age-matched healthy controls (used as controls of CLLblood, briefly as CLLcontrol), 21 peripheral blood mononuclear samples of T-PLL patients and 34 peripheral blood mononuclear samples of healthy controls (used as normal controls of T-PLL, briefly as T-PLLcontrol), and 70 CLL samples of monocytes in blood (CLLcell). The CLLcell samples include 35 CLL samples treated with radiation (CLL-Ir) and 35 CLL samples not treated with radiation (CLL-Un). 35 CLL-Ir include 17 CLL-Ir-N3 samples (treated with nutlin-3) and 18 CLL-Ir-Un (not treated with nutlin-3) and 35 CLL-Un include 17 CLL-Un-N3 (treated with nutlin-3) and 18 CLL-Un-Un (not treated with nutlin-3).

### 3.2 An overview of miRNA M/E sites in leukemia

**FIGURE 1:**
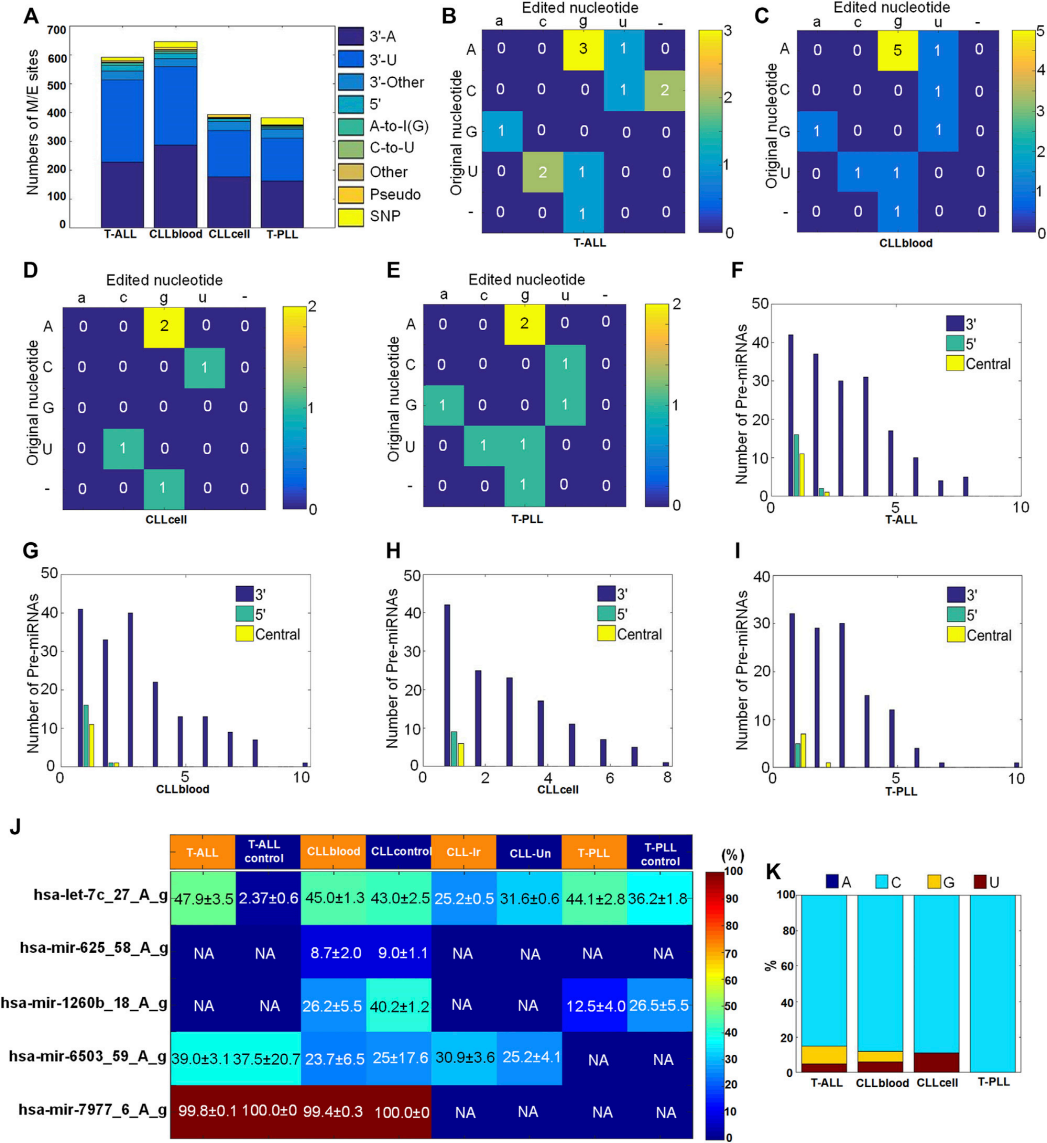
Summary of miRNA M/E sites identified in three subtypes of leukemia **(A)** The numbers of different types of miRNA M/E sites with significant editing levels in the selected samples. **(B–E)** The numbers of different types of editing sites in the central regions of miRNAs in T-ALL, CLLblood, CLLcell, and T-PLL **(F–I)** The numbers of pre-miRNAs with editing events at the 3′-end, 5′-end and central regions of mature miRNAs in T-ALL, CLLblood, CLLcell, and T-PLL, respectively **(J)** The editing levels of A-to-I editing sites in different subtypes of leukemia. CLLcell was divided as CLL-Ir and CLL-Un. The values in the matrix are mean values ± standard errors of the means. “NA” indicates not available by using the criteria that the M/E sites have significant editing levels in at least 20% samples selected. **(K)** The percentages of different types of nucleotides in 5′-editing sites identified in the selected samples.

We used the MIRME pipeline (Zheng et al., 2016) with default parameters to analyze the small RNA sequencing data from three different subtypes of leukemia. As shown in Figure 1A, we found 591 significant M/E sites in 48 T-ALL samples and four normal thymocytes samples (as shown in Supplementary Table S2), and 646 M/E sites in 21 CLL blood samples and two age-matched healthy controls (as shown in Supplementary Table S3), and 394 significant M/E sites in CLL samples from monocytes (as shown in Supplementary Table S4), and a total of 381 M/E editing sites were identified in 21 T-PLL samples and 34 healthy controls from peripheral blood monocytes (as shown in Supplementary Table S5). We found that the percentages of 3′-A and 3′-U sites were the largest in the nine different categories of M/E identified in T-ALL, CLLblood, CLLcell, and T-PLL datasets, respeatively (as shown in Figure 1A).

Next, according to the variation types of bases, the specific distribution of editing types of A-to-I, C-to-U and Other types sites in each leukemia type were further investigated. 3 A-to-I editing events, 1 C-to-U editing events and eight Other editing events were detected in T-ALL and T-ALLcontrol samples, and two insertion or deletion were also found (see Figure 1B and Supplementary Table S10). 5 A-to-I editing events, 1 C-to-U editing event and six Other editing events were detected in CLLblood and CLLcontrol samples (see Figure 1C and Supplementary Table S10). 2 A-to-I editing events, 1 C-to-U editing event and two Other editing events were detected in CLLcell samples (see Figure 1D and Supplementary Table S10). In T-PLL and T-PLLcontrol samples, a total of 2 A-to-I editing events, 1 C-to-U editing event and two Other editing events were detected (see Figure 1E and Supplementary Table S10).

Furthermore, we counted numbers of the editing events that happened on the 5′, 3′ end and central regions of the precursor miRNAs (pre-miRNAs). Interestingly, editing events occurred more frequently at the 3′ end than at the 5′ end and in the central region, however only one or two editing sites occurred at the 5′ end or central regions of miRNAs (see Figures 1F–I), which was similar to the results previously reported (Zheng et al., 2016; Wang et al., 2019). The 5′-editing sites had a preference of C in T-ALL, CLLblood, CLLcell and T-PLL samples (Figure 1K), similar to results noticed previously (Zheng et al., 2016).

We summarized 5 A-to-I editing sites in T-ALL, CLLblood, T-PLL and CLLcell samples in Figure 1J and Supplementary Table S6. One A-to-I editing site in hsa-let-7c, i.e., hsa-let-7c_27_A_g, has significantly higher editing level in T-ALL and T-PLL compared to corresponding normal controls (corrected *p* < 0.05, Mann-Whitney *U*-tests), as shown in Figure 1J and Supplementary Table S6. One of the A-to-I editing sites identified in hsa-mir-6503 (hsa-mir-6503_59_A_g) was also reported in a previous study of miRNA A-to-I editing sites in multiple cancers (Wang et al., 2017). Moreover, Gassner et al. (2020) found that the A-to-I editing site in hsa-mir-6503 occurred exclusively in CLL cell samples (7 of 45). Our results indicated that the editing level of hsa-mir-6503_59_A_g did not show severe changes in T-ALL, CLLblood and CLLcell samples.

### 3.3 PCA and clustering analysis in three subtypes of leukemia

**FIGURE 2:**
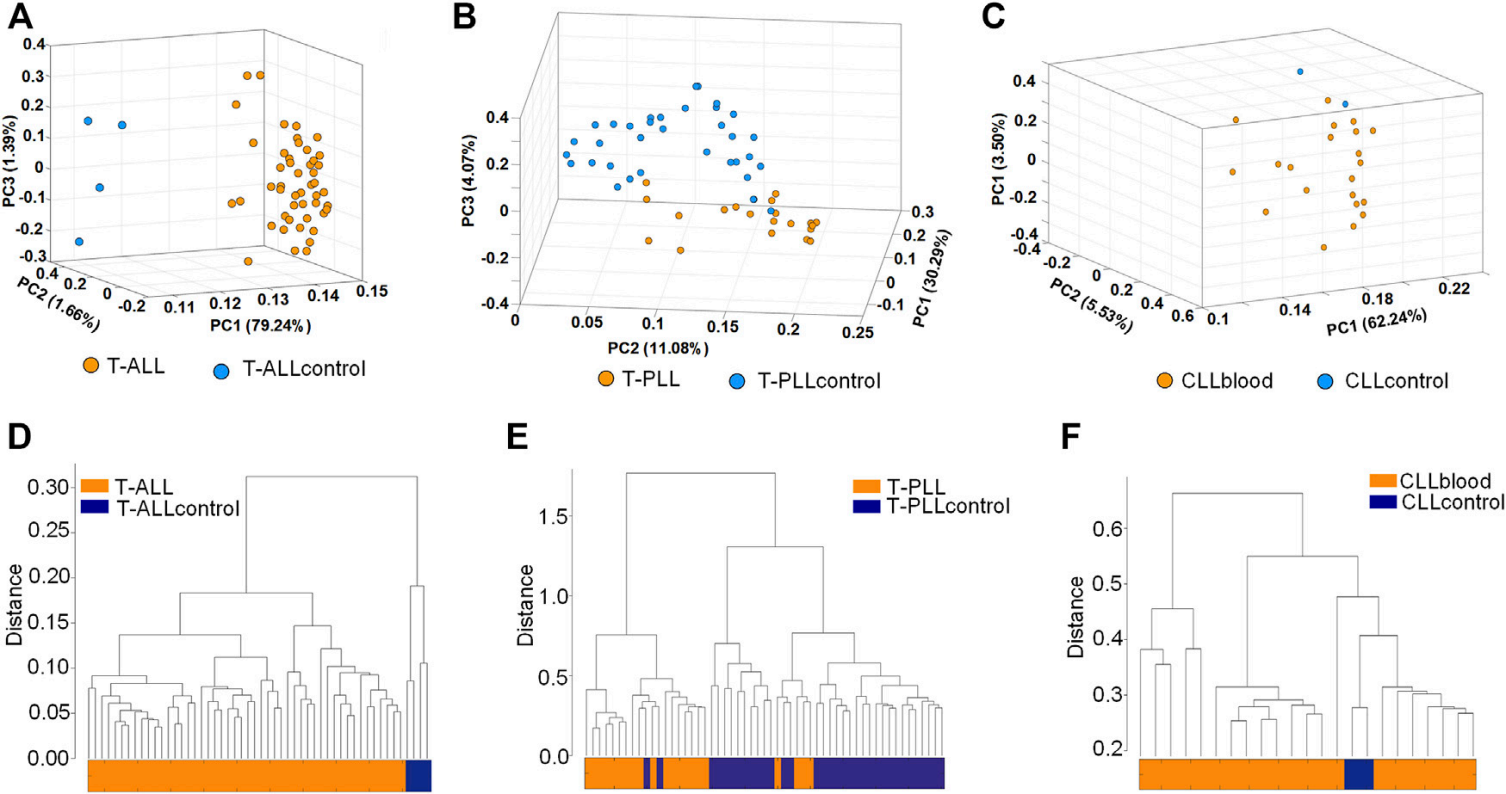
Principal component analysis and hierarchical clustering analysis **(A**–**C)** The principal component analysis of T-ALL, T-PLL and CLLblood samples, respectively. The editing levels of M/E sites were used **(D**–**F)** The hierarchical clustering of T-ALL, T-PLL and CLLblood samples, respectively. The expression levels of original miRNAs, edited miRNAs and the editing levels of the M/E sites after normalization were used.

We used the editing levels of M/E sites to perform principal component analysis (see Materials and methods). As shown in Figure 2A and Supplementary Table S11, T-ALL and control samples can be clearly distinguished in the PCA analysis. Most of the T-PLL and CLLblood samples were grouped together (Figures 2B,C, respectively). We next used the expression levels of original miRNAs and edited miRNAs, and the editing levels of the M/E sites after normalization to perform hierarchical clustering analysis. Results showed that T-ALL samples were clustered together while normal control samples were clustered together (Figure 2D). Similar to results of PCA, most T-PLL were clustered together, and six of the 55 samples were incorrectly grouped (see Figure 2E). The CLLblood samples were grouped together with the two normal controls (Figure 2F). These results suggest that tumor and normal samples have different miRNA editing profiles.

The CLL-Ir and CLL-Un samples were not separable in the principal component analysis and hierarchical clustering analysis (results not shown), probably because the CLL-Ir and CLL-Un were all CLL cell samples.

### 3.4 Identified SNPs in miRNAs

We compared the identified M/E sites with previously reported SNPs in dbSNP (v151) database and literature, and checked their editing levels. Totally, 11, 21 and 25 SNPs were identified in T-ALL, CLLblood, T-PLL and their corresponding controls, respectively (Supplementary Figures S1A, S2A, and S3A, respectively, and Supplementary Tables S16-18, respectively). Two examples of 11 SNPs in miRNAs in T-ALL were shown in Supplementary Figures S1B-E, and the editing levels of these two sites were 100% in the selected samples. Two of the 21 SNPs in CLLblood were shown in Supplementary Figures S2B-E, and the editing levels of these two sites were 100% in the two samples selected too. Two of the 25 SNPs in T-PLL were shown in Supplementary Figures S3B-E, and the editing levels of these two SNPs were 100% in the selected samples.

### 3.5 Analysis of common miRNA editing sites in three subtypes of leukemia

**FIGURE 3:**
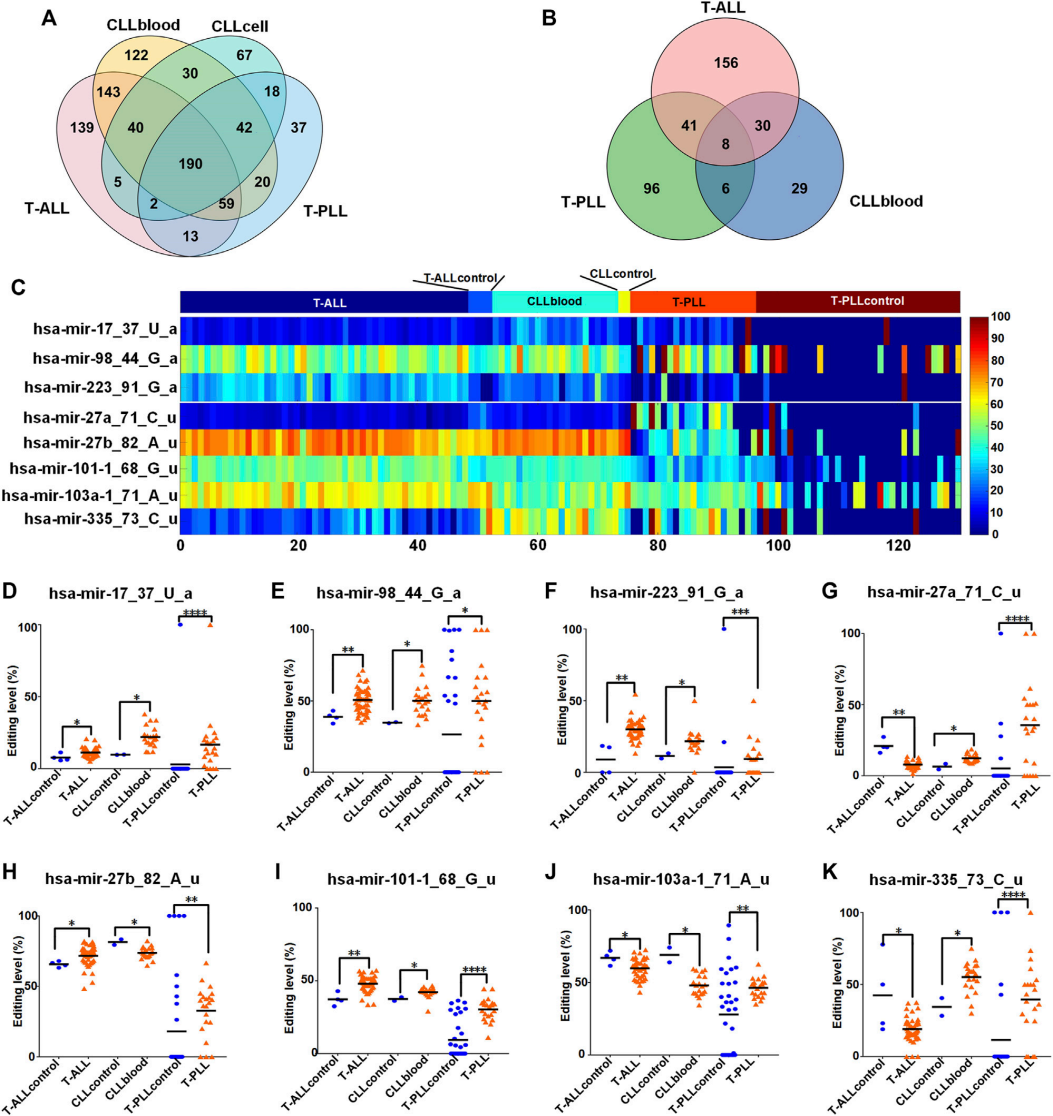
Comparisons of M/E sites in different subtypes of leukemia **(A)** The Venny diagram of miRNA editing events in T-ALL, CLLblood, CLLcell, and T-PLL leukemia samples. **(B)**The Venny diagram of M/E sites with significantly different editing levels in T-ALL, CLLblood and T-PLL compared with their corresponding controls, respectively. The M/E sites with *p* < 0.05, Mann-Whitney *U*-tests in T-ALL, CLLblood and T-PLL were compared and shown in figure **(C)** The editing levels of 8 M/E sites with significantly different present in T-ALL, CLLblood, and T-PLL samples and their normal control samples. **(D**–**K)** The comparisons of editing levels of these 8 M/E sites in each leukemia and its normal control samples. In Part **(D**–**K)**, “****“, “***“, “**“, and “*” indicate *p*-values < 0.0001, 0.001, 0.01 and 0.05, respectively, Mann-Whitney *U*-tests.

We found 190 common M/E sites in all the three leukemia subtypes (as shown in Figure 3A and Supplementary Table S12). Then, we compared the editing levels of M/E sites in three different subtypes of leukemia to their corresponding normal controls. The results showed that there were eight common M/E sites with significantly different editing levels in T-ALL, CLLblood, T-PLL samples compared to their corresponding control samples, respectively (*p* < 0.05, Mann-Whitney *U*-tests, Figures 3B,C and Supplementary Table S12).

The editing levels of these 8 M/E sites in T-ALL, CLLblood and T-PLL and their corresponding normal control samples were compared (as shown in Figures 3D–K). The editing levels of four of these 8 M/E sites (hsa-mir-17_37_U_a, hsa-mir-98_44_G_a, hsa-mir-223_91_G_a and hsa-mir-101–1_68_G_u) showed higher editing levels in the three subtypes of leukemia comparing to their control samples, respectively (Figures 3D–F,I, respectively). Two M/E sites (hsa-mir-27a_71_C_u and hsa-mir-335_73_C_u) in Figures 3G,K, respectively, also showed the same directions of changes in different subtypes of leukemia comparing to their control samples, respectively. hsa-mir-27b_82_A_u had increased editing level in T-ALL and T-PLL, but had decreased editing level in CLLblood samples (Figure 3H). hsa-mir-103a-1_71_A_u showed decreased editing level in T-ALL and CLLblood samples, but showed increased editing level in T-PLL samples (Figure 3J).

The existence of common miRNA editing sites with similar changes in their editing levels in T-ALL, CLLblood and T-PLL samples suggests that there may be common mechanism of miRNA editing in different subtypes of leukemia.

### 3.6 Relevant miRNA M/E sites in CLLcell

**FIGURE 4:**
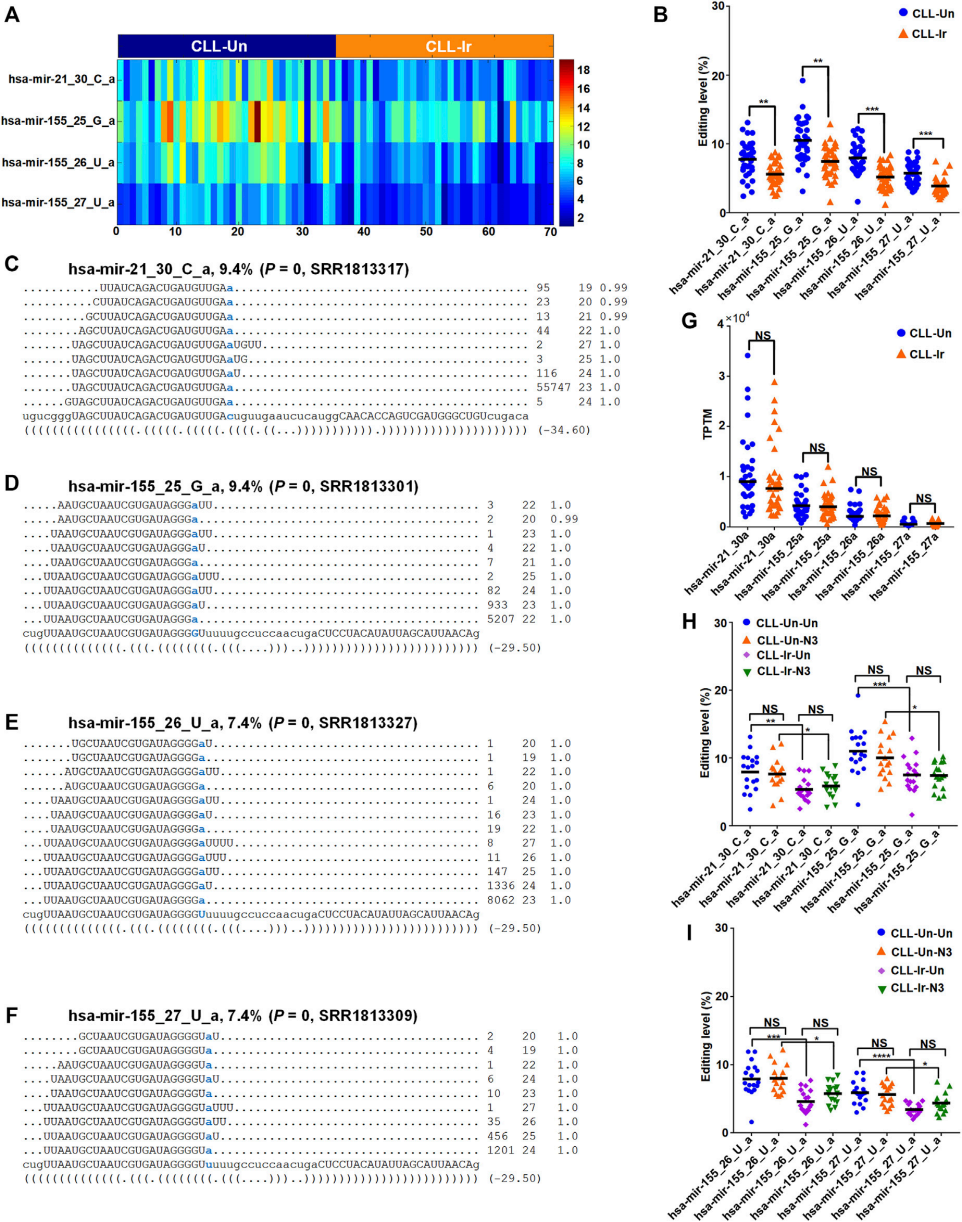
Four M/E sites with significantly different editing levels in CLL **(A)** The editing levels of this 4 M/E sites with significantly different in the 70 selected sRNA-seq profiles. **(B)** The editing levels of 4 M/E sites with significantly different present in CLL-Ir and CLL-Un samples **(C**—**F)** The detailed reads of hsa-mir-21_30_C_a in one of the CLL-Un samples (SRR1813317), hsa-mir-155_25_G_a in one of the CLL-Un samples (SRR1813301), hsa-mir-155_26_U_a in one of the CLL-Un samples (SRR1813327), and hsa-mir-155_27_U_a in one of the CLL-Un samples (SRR1813309), respectively **(G)** The expression levels of four edited miRNAs (hsa-mir-21_30a, hsa-mir-155_25a, hsa-mir-155_26a, and hsa-mir-155_27a) in CLL-Ir and CLL-Un samples. “NS” indicates not significant, i.e., corrected *p*-value > 0.05, edgeR **(H,I)** The distributions of editing levels of these 4 M/E sites in CLL-Un-Un, CLL-Un-N3, CLL-Ir-Un, and CLL-Ir-N3 samples. In Part **(B)**, and **(H,I)**, “****“, “***“, “**“, and “*” indicate corrected *p*-values < 0.0001, 0.001, 0.01 and 0.05, respectively; and “NS” indicates not significant, i.e., corrected *p*-value > 0.05, Mann-Whitney *U*-tests. In Part **(C–F)**, the edited nucleotides are shown in blue. And the first number on the right indicates the frequency of the read, the second number indicates the length of the read, and the third number indicates the weight of the read on this locus as calculated by the cross-mapping correction algorithm.

We compared the editing levels of M/E sites in CLL-Ir and CLL-Un samples, and found that there were 4 M/E sites in CLL-Ir samples with significantly lower editing levels than CLL-Un samples (corrected *p*-values < 0.05, Mann-Whitney *U*-tests). These 4 M/E sites are hsa-mir-21_30_C_a, hsa-mir-155_25_G_a, hsa-mir-155_26_U_a, and hsa-mir-155_27_U_a respectively (as shown in Figures 4A,B and Supplementary Table S13), all of which were 3′-A editing events (as shown in Figures 4C–F). The expression levels of four miRNAs were slightly downregulated and almost not changed, respectively, in CLL-Ir samples compared with CLL-Un samples (as shown in Figure 4G).

We next compared the editing levels of these 4 M/E sites in four comparisons, i.e., CLL-Un-Un to CLL-Un-N3, CLL-Un-Un to CLL-Ir-Un, CLL-Un-N3 to CLL-Ir-N3, and CLL-Ir-Un to CLL-Ir-N3 samples, to examine the treatments of irradiation and nutlin-3 in miRNA editing levels. The results showed that the editing levels of these 4 M/E sites was significantly lower in CLL-Ir-Un samples compared with CLL-Un-Un samples, and also decreased significantly in CLL-Ir-N3 samples compared with CLL-Un-N3 samples (Figures 4H,I). These results indicated that irradiation significantly decreased the editing levels of 3′-A of hsa-mir-21-5p and hsa-mir-155-5p, which potentially resulted in the downregulation of these two oncomiRNAs after irradiation.

In the meantime, we also found that the editing levels of these 4 M/E sites had no significantly different between CLL-Un-Un samples and CLL-Un-N3 samples, and had also no significantly different between CLL-Ir-N3 samples and CLL-Ir-Un samples (Figures 4H,I). The results showed that the nutlin-3 treatment had no effect on the editing levels of miRNAs. Therefore, our result suggested that irradiation reduced the editing levels of 3′-A editing sites of hsa-mir-21-5p and hsa-mir-155-5p, while the nutlin-3 treatment had no effect on the expression of hsa-mir-21-5p and hsa-mir-155-5p.

### 3.7 Target analysis of original and edited miRNA in T-ALL

After comparing the editing levels of M/E sites in T-ALL and T-ALLcontrol samples, we found that 44 M/E sites have significantly different editing levels in T-ALL (corrected *p* < 0.05, Mann-Whitney *U*-tests, Supplementary Table S6.1). Among these M/E sites, we focused on three 5′-editing sites because these editing sites changed the seed of their corresponding miRNAs.

**FIGURE 5:**
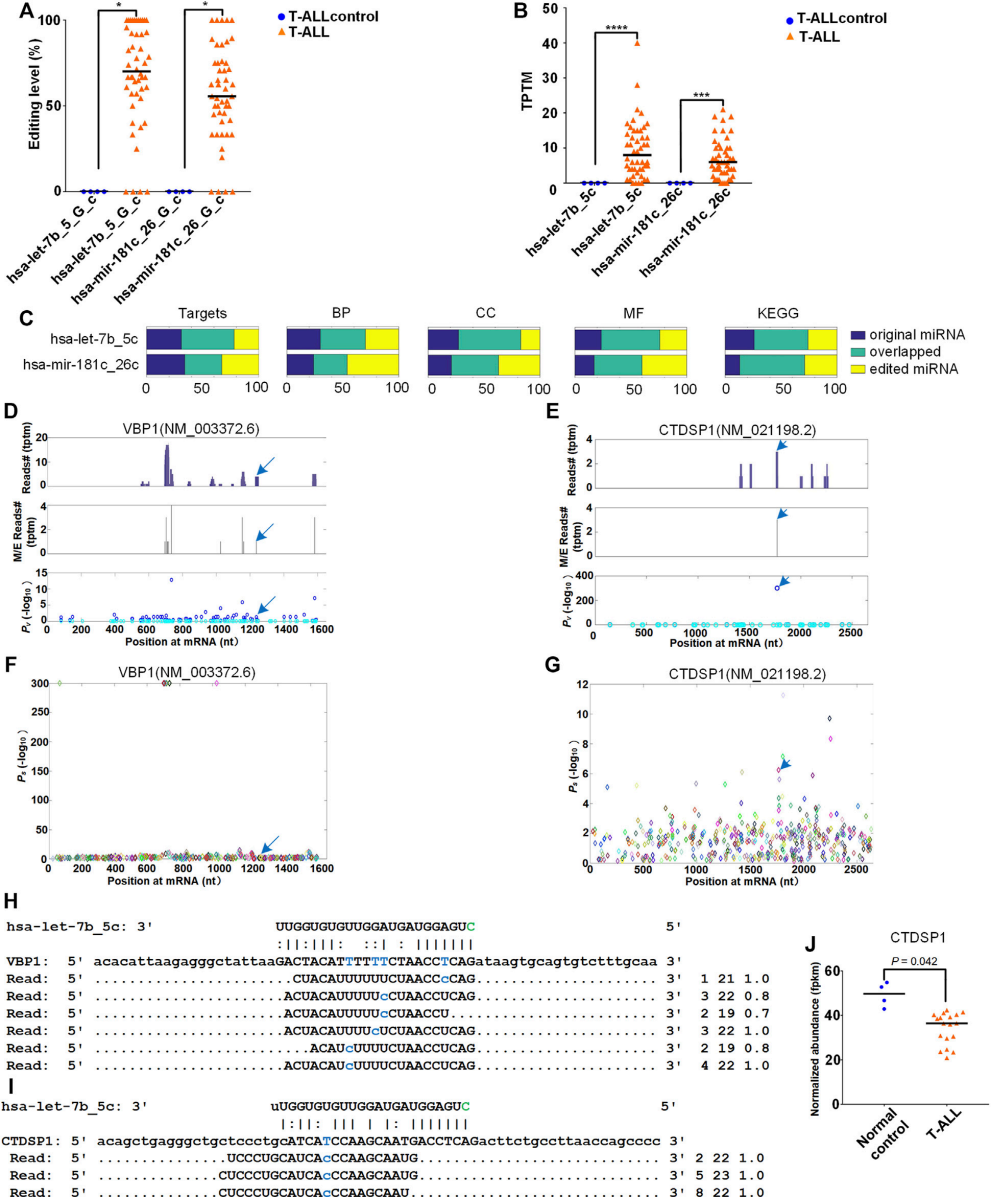
Target analysis of original and edited miRNAs in T-ALL. The detailed legend is provided on the next page. Target analysis of original and edited miRNAs in T-ALL. **(A)** The comparisons of the editing levels of two 5′-editing sites in T-ALL and T-ALLcontrol samples. “*” indicates corrected *p*-value < 0.05, Mann-Whitney *U*-tests **(B)** Comparisons of normalized expression levels (TPTM) of hsa-let-7b_5c and hsa-mir-181c_26c in the T-ALL and T-ALLcontrol samples. “****” and “***” indicate corrected *p*-values < 0.0001 and 0.001, respectively, edgeR **(C)** The comparisons of targets, GO terms, and KEGG pathways of original and two 5′-edited miRNAs. The GO terms Biological Process (BP), Cellular Component (CC) and Molecular Function (MF) are shown separately **(D,E)** The distributions of PAR-CLIP reads on VBP1 (NM_003372.6), CTDSP1 (NM_021198.2), respectively. The peaks pointed by blue arrows were the complementary sites of hsa-let-7b_5c **(F,G)** The identified M/E sites and their *P*_*s*_ values on VBP1 (NM_003372.6), CTDSP1 (NM_021198.2), respectively. The sites pointed by blue arrows were the complementary sites of hsa-let-7b_5c **(H,I)** The details of complementary sites of hsa-let-7b_5c and PAR-CLIP reads on VBP1 (NM_003372.6), CTDSP1 (NM_021198.2), respectively. In part **(H)** to **(I)**, the T-to-C nucleotides are shown in blue on mRNAs and PAR-CLIP sequencing reads. The green Cytosine on 5′ end of hsa-let-7b_5c is the additional cytosine introduced by the RNA editing event, hsa-let-7b_5c. And the first number on the right indicates the number of raw sequencing reads, the second number indicates the length of the reads, and the third number indicates the weight of the reads on this locus as calculated by the cross-mapping correction algorithm. **(J)** The normalized abundance of CTDSP1 in the samples of normal T cells compared with the samples of T-ALL patients. The multiple test corrected *p*-value was calculated by edgeR.

We firstly examined two 5′-editing sites (hsa-let-7b_5_G_c and hsa-mir-181c_26_G_c) with increased editing levels in T-ALL (corrected *p* < 0.05, as shown in Figure 5A and Supplementary Table S14). The expression levels of the two edited miRNAs (hsa-let-7b_5c and hsa-mir-181c_26c) were significantly higher in T-ALL samples than T-ALLcontrol samples (corrected *p* < 0.0001 and 0.001, edgeR, Figure 5B).

Next, we used the MiCPAR algorithm (MiRNA target prediction using Corrected PAR-CLIP) (Zheng, 2018) to identify targets of the original and edited miRNAs (Supplementary Table S7 and Supplementary Table S8). As shown in Figure 5C, the target genes of edited miRNAs changed dramatically compared to those of original miRNAs, and only small portions of targets of original and edited miRNAs were overlapped (Supplementary Table S14). We also analyzed the enriched GO terms and KEGG pathways for the targets of the original and edited miRNAs. The enrichment analysis of Gene Ontology and KEGG pathway (Kanehisa and Goto, 2000; Kanehisa, 2019, 2021) showed that edited miRNAs acquired many different GO terms and KEGG pathways (Figure 5C).

We focused on the analysis of the 5′-editing on hsa-let-7b because it is normally regarded as a tumor suppressor (Wu et al., 2020; Dong et al., 2021). After comparing the targets of original and edited miRNA, we identified 262 new targets for edited hsa-let-7b, i.e., hsa-let-7b_5c. Among the 262 newly gained targets of hsa-let-7b_5c, duplicate genes and non-coding genes were removed and targets with at least 10 PAR-CLIP reads were kept for further analysis, included VBP1 (NM_003372.6, VHL-binding protein 1) and CTDSP1 (NM_021198.2, CTD small phosphatase 1) respectively. The distributions of PAR-CLIP reads on VBP1 and CTDSP1 in Figures 5D,E indicated that there were clear accumulation of PAR-CLIP reads at the complementary sites of hsa-let-7b_5c. The identified miRNA complementary sites and their *P*_*s*_ values on VBP1 and CTDSP1 were shown in Figures 5F,G, respectively. The details of complementary sites of hsa-let-7b_5c and PAR-CLIP reads on VBP1 and CTDSP1 were also shown in Figures 5H,I. CTDSP1 is downregulated in T-ALL (as shown in Figure 5J and Supplementary Table S14). However, because the sample size of the gene expression data set used is very limited, further studies are needed to verify the expression of CTDSP1 in T-ALL.

**FIGURE 6:**
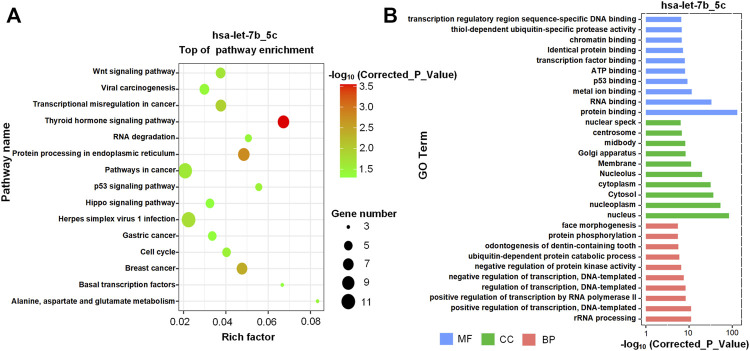
The enriched KEGG pathways and GO terms of new targets of hsa-let-7b_5c **(A)** The enriched KEGG pathways of the new targets of hsa-let-7b_5c. **(B)** The most significant GO terms of the new targets of hsa-let-7b_5c. The GO terms with the smallest corrected *p*-values in the three major GO categories, i.e., Molecular Function (MF), Cellular Component (CC) and Biological Process (BP), were presented respectively.

To understand the potential functional impact of higher expression of hsa-let-7b_5c in T-ALL, the enriched KEGG pathway analysis was performed for the 262 new targets of hsa-let-7b_5c with KOBAS (v3) (Xie et al., 2011) (Supplementary Table S15). The most significant pathways included “protein processing in endoplasmic reticulum”, “pathways in cancer” and “breast cancer” (Figure 6A). And enriched GO terms included “protein binding”, “nucleus”, and “positive regulation of transcription, DNA-templated” (see Figure 6B and Supplementary Table S15). In the same way, the enrichment analysis of GO terms and KEGG pathway of new targets of hsa-mir-181c_26c were shown in Supplementary Figures S4 and Supplementary Table S19. The enriched pathways of new targets of hsa-mir-181c_26c included “pathways in cancer” and “cell cycle” (Supplementary Figures S4A). And the enriched GO terms of new targets of hsa-mir-181c_26c included “protein binding”, “nucleoplasm”, and “positive regulation of transcription by RNA polymerase II” (Supplementary Figures S4B).

Secondly, the editing level of one 5′-editing site (hsa-let-7a-2_4_U_c) was reduced in T-ALL samples compared with T-ALLcontrol samples (corrected *p* < 0.05, Mann-Whitney *U*-test, Supplementary Figures S5). Subsequently, we compared the targets of the original and edited hsa-let-7a-5p (Supplementary Table S7 and Supplementary Table S8), and the enriched GO terms and KEGG pathways of targets of the original and edited hsa-let-7a-5p (Supplementary Figures S5C and Supplementary Table S20). The results indicated that the 5′-editing events of hsa-let-7a-5p also introduced many new targets and new functions, in terms the new GO terms and KEGG pathways (Supplementary Figures S5C). The enriched KEGG pathway of the new targets of hsa-let-7a-2_4c included “protein processing in endoplasmic reticulum” and “pathways in cancer” (Supplementary Figures S5D and Supplementary Table S20). And enriched GO terms included “protein binding”, “nucleus”, and “positive regulation of transcription, DNA-templated” (Supplementary Figures S5E). However, the expression levels of the edited miRNAs (hsa-let-7a-2_4c) was significantly increased in T-ALL compared to T-ALLcontrol samples (corrected *p* < 0.05, edgeR) which was opposite to its editing levels (Supplementary Figures S5A,B and Supplementary Table S20). We speculated that they might be some unknown mechanism that regulated their expression levels.

### 3.8 Target analysis of original and edited miRNA in T-PLL

We found that 220 M/E sites had significantly different editing levels (corrected *p* < 0.05, Mann-Whitney *U*-tests) in T-PLL samples compared to normal controls (Supplementary Table S6.4). We then focused on four 5′-editing sites (hsa-let-7f-1_6_G_c, hsa-let-7f-2_7_A_c, hsa-mir-103a-2_47_A_c, and hsa-mir-181a-1_23_G_c) and one C-to-U editing site (hsa-mir-3653_42_C_u), because these sites changed the seed regions of the mature miRNAs (Supplementary Figures S6A,B). The expression levels of the edited miRNAs corresponding these five sites have upregulated trends in T-PLL samples than T-PLLcontrol samples (corrected *p* > 0.05, edgeR, Supplementary Figures S6C,D and Supplementary Table S21). These five edited miRNAs acquired many novel targets compared to those of original miRNAs (Supplementary Figure S6E). And the enriched GO terms and KEGG pathways of the targets of these five edited miRNAs were also very different from those of the original miRNAs, suggesting that these editing events severely changed the functions of the corresponding mature miRNAs.

## 4 Discussion

After analyzing sRNA-seq profiles of T-ALL, CLLblood, CLLcell and T-PLL and their corresponding control samples, 591, 646, 394 and 381 M/E sites with significant editing level were identified, respectively. As shown in Figure 1A, we can see that most editing events of miRNAs occur at the 3-’end of mature miRNAs, which is consistent with the results of previous studies (Zheng et al., 2016; Wang et al., 2019; Guo et al., 2022). Although stringent criteria were used in our analysis to control possible false positive predictions, there still exist the possibility of false positives in the identified M/E sites which may be improved with biochemical experiments.

Although a few studies have systematically investigated the relationship between miRNAs in different leukemia subtypes, our study is the first show for the distribution of different types of miRNA editing events in three subtypes of leukemia, significantly increasing our understanding of miRNA editing events in leukemia. In the clustering and principle component analysis, the results showed that leukemia samples clustered together while normal control samples clustered together, as seen in Figure 2. Therefore, the result provides an important basis for us to use the editing level of miRNAs to distinguish between disease and normal samples, and may be an important way to detect disease. Because of the limited sample size of CLLcontrol, the results in Figures 2C,F should be re-evaluated when more control samples are available.

Next, we found 190 common M/E sites in three subtypes of leukemia. Then we found that there were eight common M/E sites with significantly different editing levels in T-ALL, CLLblood, T-PLL and their corresponding control samples, as shown in Figure 3B. All of these eight sites are 3′-editing sites, with 3′-A sites (hsa-mir-17_37_U_a, hsa-mir-98_44_G_a, hsa-mir-223_91_G_a) and five 3′-U sites (hsa-mir-27a_71_C_u, hsa-mir-27b_82_A_u, hsa-mir-101–1_68_G_u, hsa-mir-103a-1_71_A_u, and hsa-mir-335_73_C_u). Previous researches showed that 3′-NTA additions increase stability of miRNAs (Burroughs et al., 2010; D’Ambrogio et al., 2012; Katoh et al., 2009) in contrast to uridylation, which promotes degradation (Kim et al., 2015). These suggest that these 8 M/E sites may affect the expression levels of their corresponding mature miRNAs.

Hsa-mir-21-5p is a carcinogenic miRNA and overexpressed in many human malignancies such as acute myeloid leukemia (AML), lung, brain and breast cancer (Si et al., 2007; Bourguignon et al., 2009; Liu et al., 2021). Similarly, many studies reported overexpression of hsa-mir-155-5p in most solid and hematological malignancies (Liu et al., 2014; Robertson et al., 2014; Baba et al., 2016; Zeng et al., 2016; Pagotto et al., 2018, 2019; Soltani et al., 2021), B-cell chronic lymphocytic leukemia (Cui et al., 2013; Ferrajoli et al., 2014; Due et al., 2016; Pagotto et al., 2019), and CLL Ferrajoli et al. (2014). The upregulation of hsa-mir-155-5p was associated with subsequent risk of CLL (Balatti et al., 2020). After irradiation treatment, the 3′-A editing of hsa-mir-21-5p and hsa-mir-155-5p showed significant decreased editing levels compared to controls (Figure 4B). Existing evidence indicated that 3′-NTA of miRNA stabilized miRNA (Burroughs et al., 2010; D’Ambrogio et al., 2012; Katoh et al., 2009). Probably, due to decreased level of 3′-A, the expression levels of hsa-mir-21-5p and hsa-mir-155-5p were slightly downregulated and almost not changed in CLL-Ir compared to controls, respectively (Figure 4G). However, chemical treatments of nutlin-3 have no effects to the 3′-A of hsa-mir-21-5p and hsa-mir-155-5p (Figures 4H, 4U). In summary, these results suggest that irradiation is a preferred treatment of CLL, compared to chemical treatment of nutlin-3, because of its repressive effect on two oncomiRNAs, i.e., hsa-mir-21-5p and hsa-mir-155-5p.

When we compared the editing level of M/E sites in T-ALL and T-ALLcontrol samples, we found that three 5′-editing sites (hsa-let-7b_5_G_c, hsa-mir-181c_26_G_c, and hsa-mir-486–1_3_A_c) had significantly higher editing levels in T-ALL compared to normal controls (as shown in Figure 5A and Supplementary Table S14), and the expression levels of the corresponding edited miRNAs were also higher than controls (Figure 5B). Hsa-let-7b-5p normally exists as a tumor suppressor in previous studies (Wu et al., 2020; Dong et al., 2021). We compared the targets of the original hsa-let-7b-5p and the edited hsa-let-7b-5p (hsa-let-7b_5c). We found that hsa-let-7b_5c acquired 292 additional targets, including VBP1 and CTDSP1. Recently, some studies demonstrated that CTDSP1 inhibited the proliferation, migration and invasion of cancer cells by inhibiting the dephosphorylation of TWIST and AKT, suggesting a tumor suppressor role of CTDSP1 (Rallabandi et al., 2020; Yang X. et al., 2021). Furthermore, a study showed the role of CTDSP1 in cell cycle arrest during the G1/S transition through c-Myc-mediated dephosphorylation of retinoblastoma protein (RB) (Liu et al., 2016; Wang et al., 2016). Another study showed that high CTDSP1 levels were associated with better survival (Bracci et al., 2012). The gene expression of CTDSP1 was significantly lower (corrected *p* < 0.05, edgeR) in T-ALL patients samples than normal control (Figure 5J). So the downregulation of CTDSP1 in T-ALL may result from higher editing level of hsa-let-7b_5_G_c and higher expression level of hsa-let-7b_5c in T-ALL.

In summary, we reported the first view of miRNA editing in three subtypes of leukemia which increased our understanding of miRNA editing events in leukemia. More research is needed in the future to clarify the relationship between deregulated miRNA editing in leukemia and the pathogenesis of leukemia.

In addition to miRNAs, other types of non-coding RNAs, such as tRNA derived small non-coding RNAs (Veneziano et al., 2019) and snoRNAs (Zhou et al., 2017), might be edited or modified in leukemia. It is interesting to explore the editing and/or modification events in these RNAs too. Furthermore, PAR-CLIP profiles of tissues or cell lines related to leukemia, such as the PAR-CLIP profile of primary effusion B cell lymphoma cell lines (Gottwein et al., 2011), could be used to improve the identification of targets of edited miRNAs in leukemia.

## Data Availability

The original contributions presented in the study are included in the article/Supplementary Material, further inquiries can be directed to the corresponding author.
