# Supplementary material for: Identification of microRNA editing sites in three subtypes of leukemia
Source: Front Mol Biosci. 2022 Nov 14;9:1014288. doi: 10.3389/fmolb.2022.1014288 (PMC9702332; doi:10.3389/fmolb.2022.1014288)
Supplement: Supplementary file 2 [file DataSheet3.pdf]

Supplementary Material

1 SUPPLEMENTARY TABLES AND FIGURES

1.1 Tables

There are 21 supplementary tables as following:

Supplementary Table S1 | The 52 sRNA-seq profiles of T-ALL and T-ALLcontrol analyzed in this study (Table S1.1). The 70 sRNA-seq profiles of CLLcell and 23 sRNA-seq profiles of CLLblood and CLLcontrol analyzed in this study (Table S1.2). The 55 sRNA-seq profiles of T-PLL and T-PLLcontrol analyzed in this study (Table S1.3). The 11 PAR-CLIP sequencing profiles used to predict targets for original and edited miRNAs (Table S1.4).

Supplementary Table S2 | The 591 editing sites identified in T-ALL.

Supplementary Table S3 | The 646 editing sites identified in CLLblood.

Supplementary Table S4 | The 394 editing sites identified in CLLcell.

Supplementary Table S5 | The 381 editing sites identified in T-PLL.

Supplementary Table S6 | The comparisons of the editing levels of 591 M/E sites between the T-ALL and T-ALLcontrol sRNA-seq profiles (Table S6.1). The comparisons of the editing levels of 646 M/E sites between the the CLLblood and CLLcontrol sRNA-seq profiles (Table S6.2). The comparisons of the editing levels of 394 M/E sites between the CLL-Ir and CLL-Un sRNA-seq profiles (Table S6.3). The comparisons of the editing levels of 381 M/E sites between the T-PLL and T-PLLcontrol sRNA-seq profiles (Table S6.3).

Supplementary Table S7 | The targets of 2 selected original miRNAs with significantly increased editing level in T-ALL samples (Table S7.1). The targets of 1 selected original miRNAs with significantly decreased editing level in T-ALL samples (Table S7.2). The targets of 4 selected original miRNAs with significantly increased editing level in T-PLL samples (Table S7.3).

Supplementary Table S8 | The targets of 2 selected edited miRNAs with significantly increased editing level in T-ALL samples (Table S8.1). The targets of 1 selected edited miRNAs with significantly decreased editing level in T-ALL smaples (Table S8.2). The targets of 5 selected edited miRNAs with significantly increased editing level in T-PLL samples (Table S8.2).

Supplementary Table S9 | The GO and KEGG analysis results of miRNAs targets.

Supplementary Table S10 | Source data of Figure 1.

Supplementary Table S11 | Source data of Figure 2.

Supplementary Table S12 | Source data of Figure 3.

Supplementary Table S13 | Source data of Figure 4.

Supplementary Table S14 | Source data of Figure 5.

Supplementary Table S15 | Source data of Figure 6.

Supplementary Table S16 | Source data of Figure S1.

Supplementary Table S17 | Source data of Figure S2.

Supplementary Table S18 | Source data of Figure S3.

Supplementary Table S19 | Source data of Figure S4.

Supplementary Table S20 | Source data of Figure S5.

Supplementary Table S21 | Source data of Figure S6.

1.2 Figures

There are 6 supplementary figures.

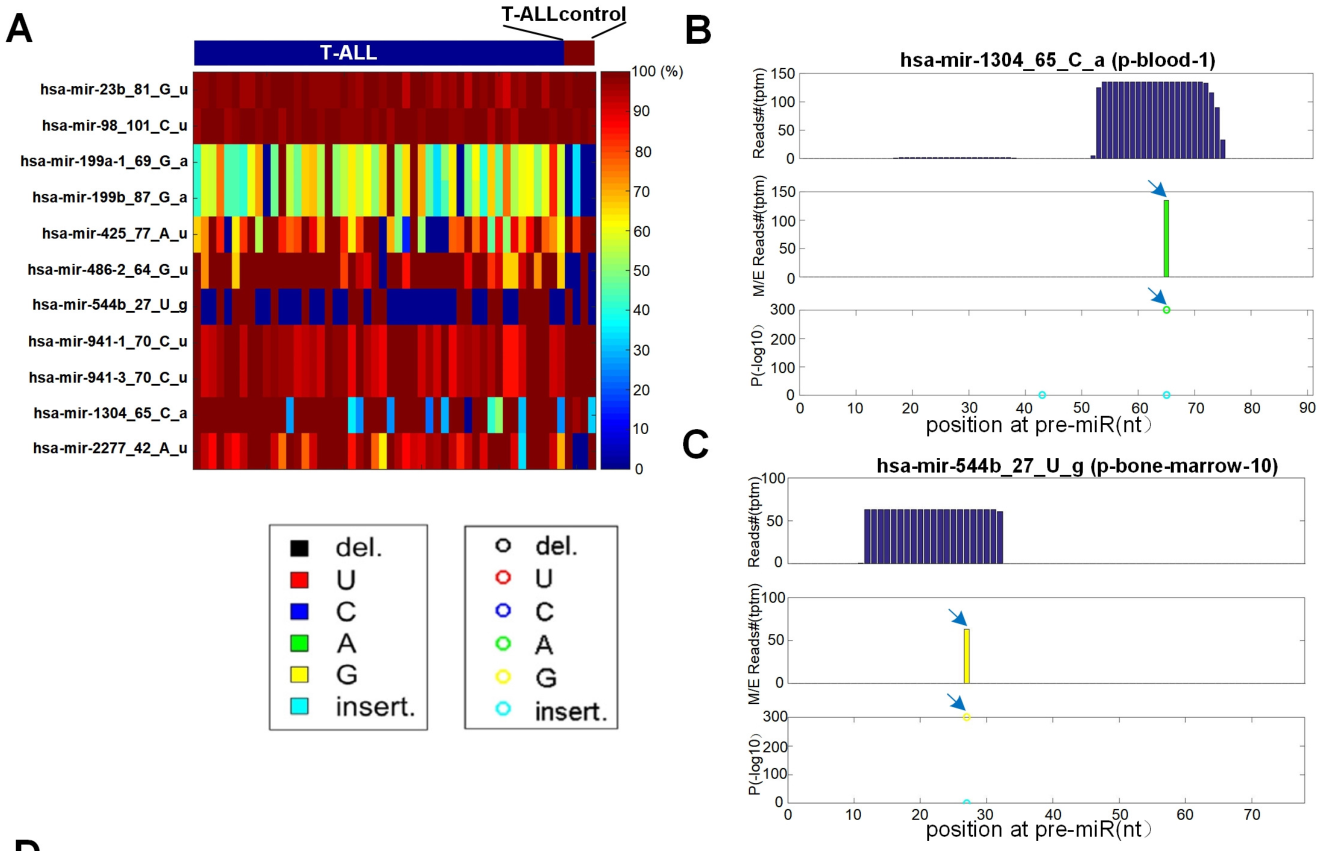

D

hsa-mir-1304_65_C_a, 100% (*P*=0,p-blood-1) ~ rs2155248(G/A/C/T)

```
.....................................................CUCACUGUAGCaUCGAACCCCU................ 263  22  0.81
.....................................................CUCACUGUAGCaUCGAACCCC................. 4    21  0.82
.....................................................CUCACUGUAGCaUCGAACCC.................. 7    20  0.76
....................................................UCUCACUGUAGCaUCGAACCCCUG............... 3    24  1.0
....................................................UCUCACUGUAGCaUCGAACCCCU................ 282  23  1.0
....................................................UCUCACUGUAGCaUCGAACCCC................. 402  22  1.0
....................................................UCUCACUGUAGCaUCGAACCC.................. 366  21  1.0
....................................................UCUCACUGUAGCaUCGAACC................... 149  20  1.0
....................................................UCUCACUGUAGCaUCGAAC.................... 9    19  0.96
...................................................AUCUCACUGUAGCaUCGAACCCCU................ 6    24  1.0
...................................................AUCUCACUGUAGCaUCGAACCCC................. 2    23  1.0
...................................................AUCUCACUGUAGCaUCGAACCC.................. 5    22  1.0
...................................................AUCUCACUGUAGCaUCGAACC................... 3    21  1.0
...................................................AUCUCACUGUAGCaUCGAAC.................... 6    20  0.98
..................................................CAUCUCACUGUAGCaUCGAACCC.................. 8    23  1.0
..................................................CAUCUCACUGUAGCaUCGAACC................... 5    22  1.0
aaacacuugagcccagcggUUUGAGGCUACAGUGAGAUGUGauccugccacaUCUCACUGUAGCCUCGAACCCCugggcucaagugauuca
...((((((((((((.(((((((((((((((((((((((((.......))))))))))))))))))))))))).)))))))))))))..... (-73.40)
```

E

hsa-mir-544b_27_U_g, 100% (*P*=0,p-bone-marrow-10) ~rs10934682(T/G)

```
...........AAAAUGCAGAAUCCAgUUCUG.............................................. 122 21 1.0
..........UAAAAUGCAGAAUCCAgUUCU............................................... 3   21 1.0
ggaauuuuguuaaaaugcagaauccauuucuguagcucugagacuagACCUGAGGUUGUGCAUUUCUAAcaaagugcc
((.((((((((((((((((.((((((..(((((..(...)..)).)))..)).)))).))))))).))))))))).)) (-23.50)
```

Figure S1. The details of 11 SNPs identified in miRNAs of T-ALL patients. **(A)** The editing/mutation levels of the 11 SNPs sites in the 52 selected sRNA-seq profiles. **(B)** The MiRME map of hsa-mir-1304 in one of the T-ALL samples (p-blood-1). **(C)** The MiRME map of hsa-mir-544b in one of the T-ALL samples (p-bone-marrow-10). **(D)** The details of hsa-mir-1304_65_C_a in p-blood-1. **(E)** The details of hsa-mir-544_27_U_g in p-bone-marrow-10. In Part **(D)** – **(E)**, the edited nucleotides are shown in blue. And the first number on the right indicates the number of raw sequencing reads, the second number indicates the length of the reads, and the third number indicates the weight of the reads on this locus as calculated by the cross-mapping correction algorithm.

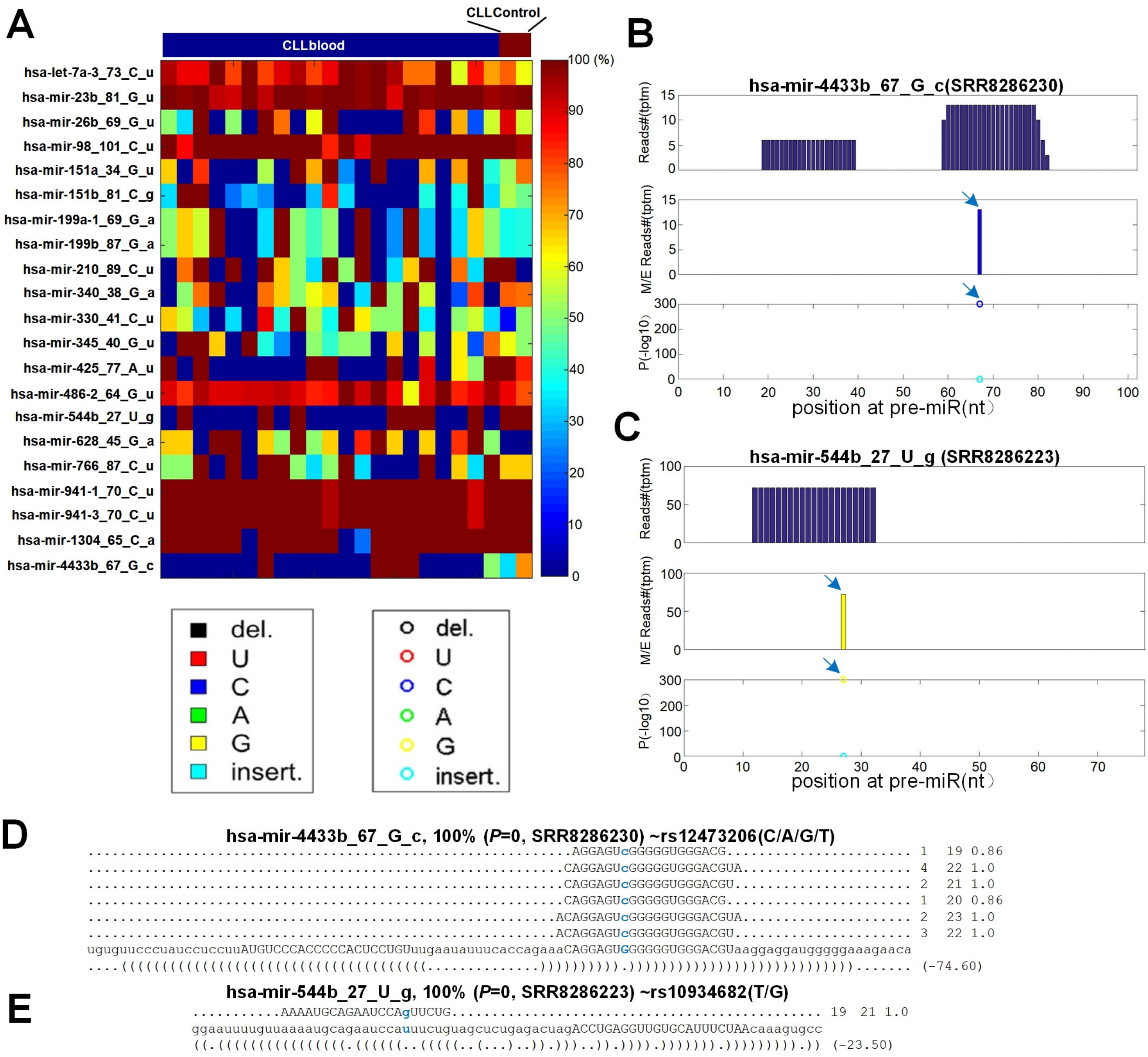

Figure S2. The details of 21 SNPs identified in miRNAs of CLLblood sample. **(A)** The editing/mutation levels of the 21 SNPs sites in the 23 selected sRNA-seq profiles. **(B)** The MiRME map of hsa-mir-4433b in one of the CLLblood samples (SRR8286230). **(C)** The MiRME map of hsa-mir-544b in one of the CLLblood samples (SRR8286223). **(D)** The details of hsa-mir-4433b_67_G_c in SRR8286230. **(E)** The details of hsa-mir-544_27_U_g in SRR8286223. In Part **(D)** – **(E)**, the edited nucleotides are shown in blue. And the first number on the right indicates the number of raw sequencing reads, the second number indicates the length of the reads, and the third number indicates the weight of the reads on this locus as calculated by the cross-mapping correction algorithm.

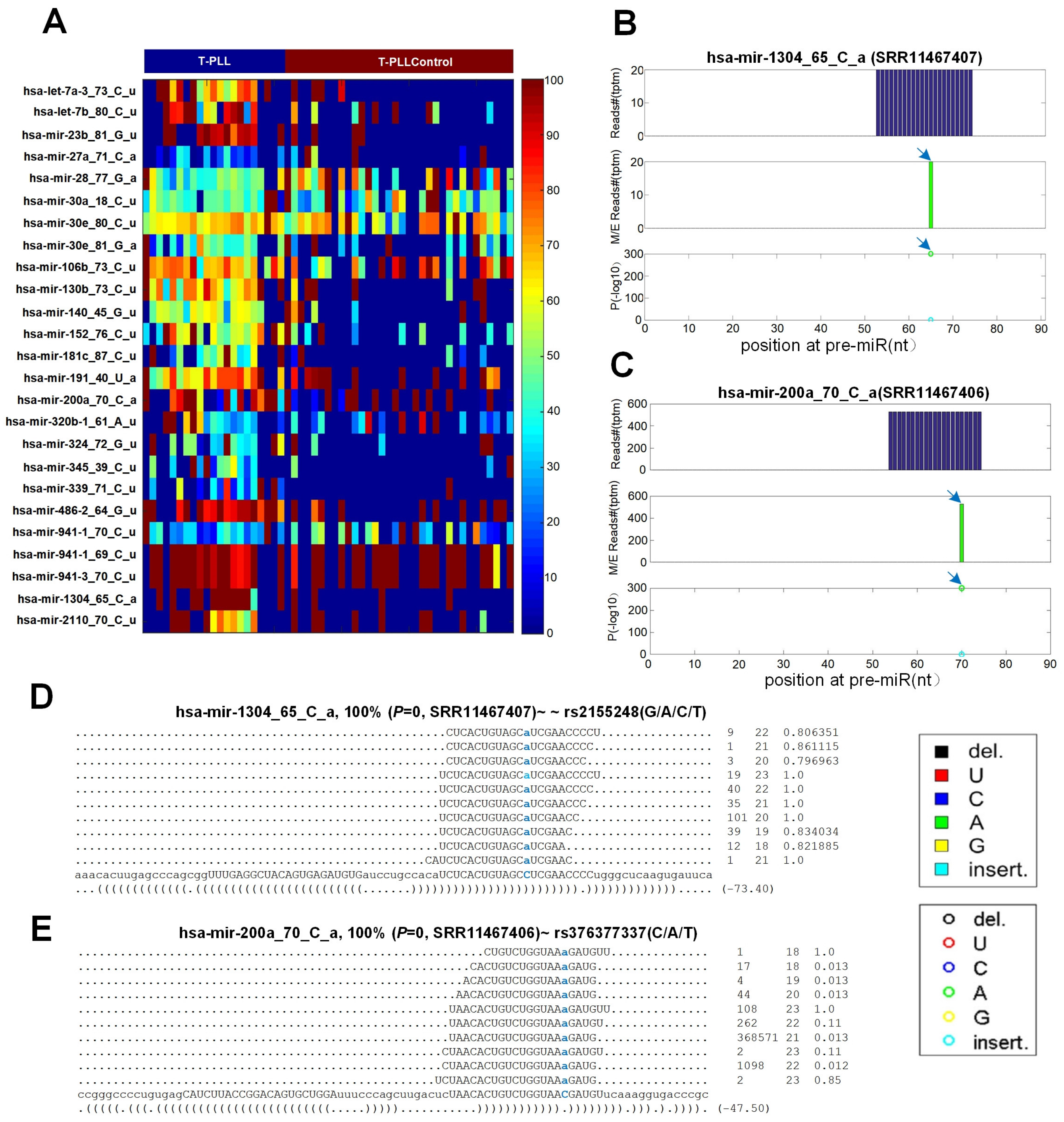

Figure S3. The details of 25 SNPs identified in miRNAs of T-PLL patients. **(A)** The editing/mutation levels of the 25 SNPs sites in the 55 selected sRNA-seq profiles. **(B)** The MiRME map of hsa-mir-1304 in one of the T-PLL samples (SRR11467407). **(C)** The MiRME map of hsa-mir-200a in one of the T-PLL samples (SRR11467406). **(D)** The details of hsa-mir-1304_65_C_a in SRR11467407. **(E)** The details of hsa-mir-200a_70_C_a in SRR11467406. In Part **(D)** – **(E)**, the edited nucleotides are shown in blue. And the first number on the right indicates the number of raw sequencing reads, the second number indicates the length of the reads, and the third number indicates the weight of the reads on this locus as calculated by the cross-mapping correction algorithm.

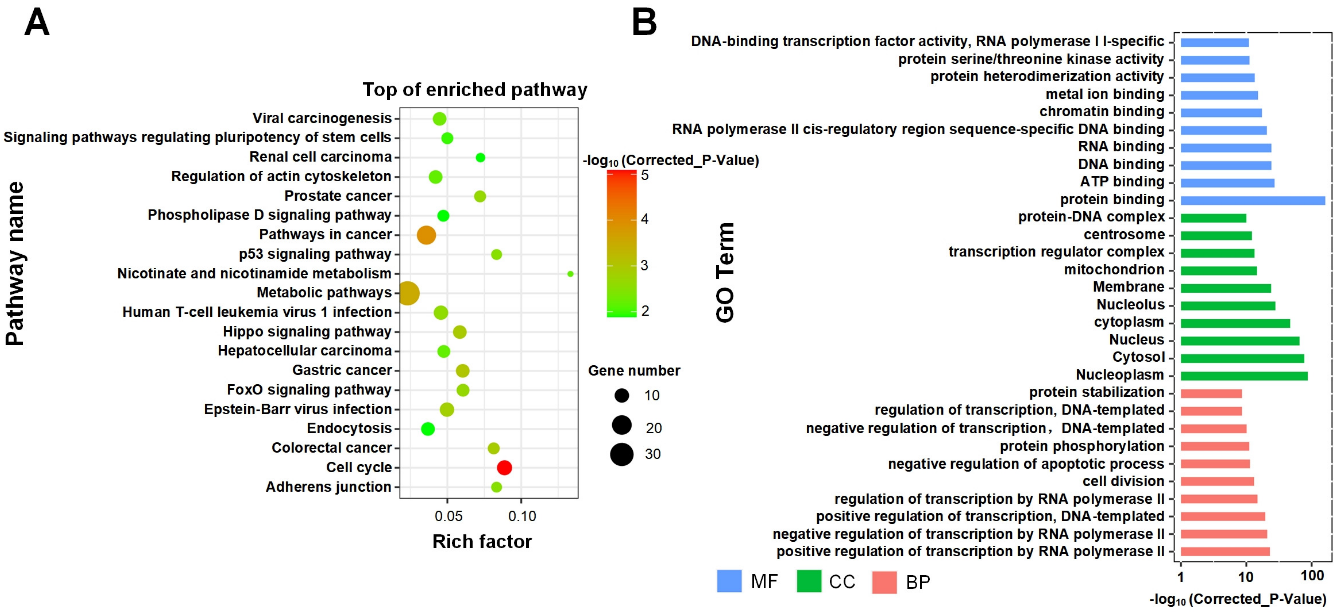

Figure S4. The enriched KEGG pathways and GO terms of new targets of hsa-mir-181c_26c. **(A)** The enriched KEGG pathways of the new targets of hsa-mir-181c_26c. **(B)** The GO terms of the new targets of hsa-mir-181c_26c. The GO terms with the smallest corrected P-values in the three GO categories, i.e., Molecular Function (MF), Cellular Component (CC) and Biological Process (BP), were presented respectively.

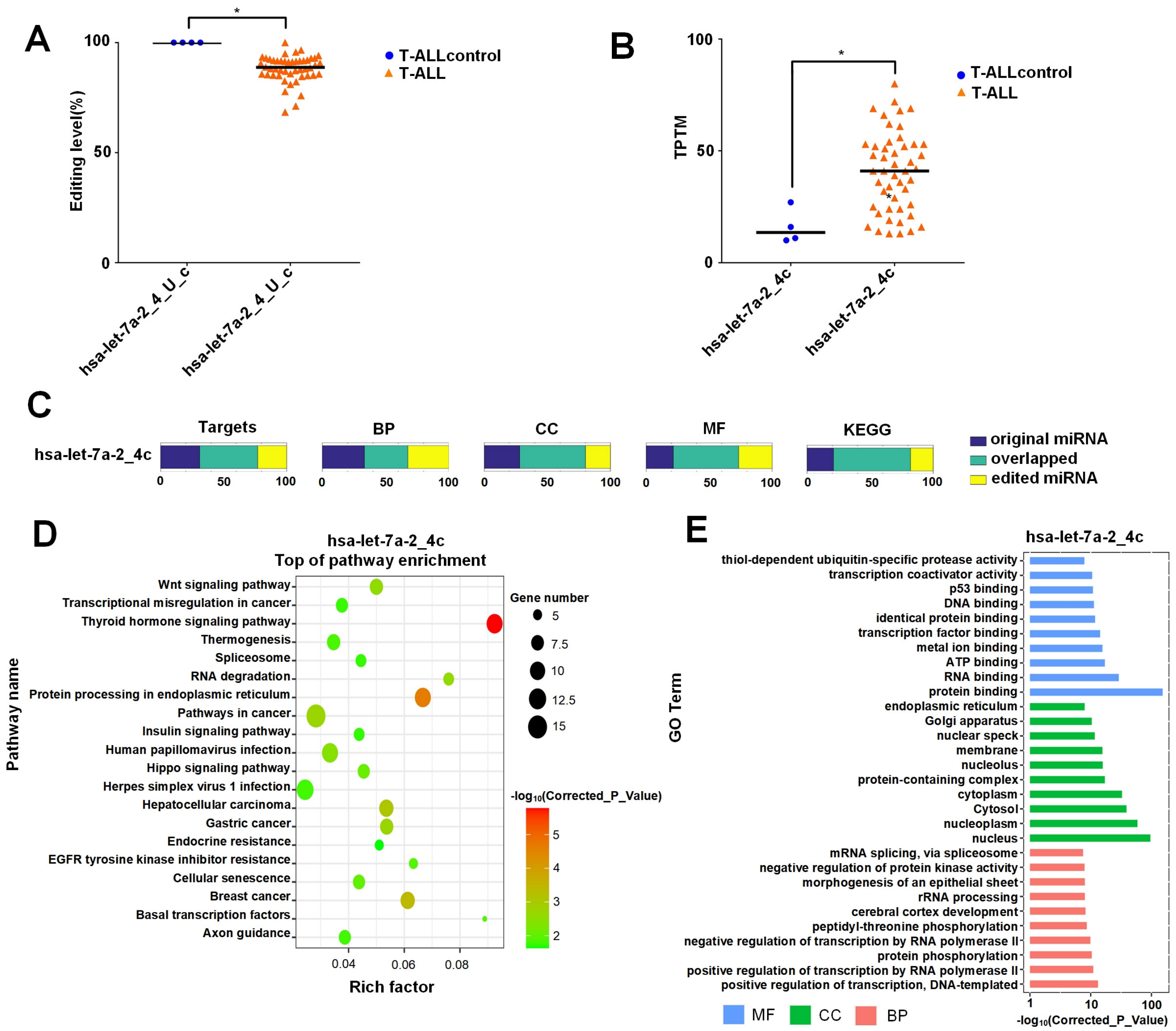

Figure S5. Target analysis of original and edited miRNAs in T-ALL. **(A)** The comparisons of the editing level of one 5'-editing site (hsa-let-7a-2_4_U_c) in T-ALL and T-ALLcontrol samples. "*" indicates corrected P-value < 0.05, Mann-Whitney U-test. **(B)** The comparisons of normalized expression level (TPTM) of hsa-let-7a-2_4c in the T-ALL and T-ALLcontrol samples. "*" indicates corrected P-value $<$ 0.05, edgeR. **(C)** The comparisons of targets, GO terms, and KEGG pathways of original and 5'-edited hsa-let-7a-2_4c. The GO terms Biological Process (BP), Cellular Component (CC) and Molecular Function (MF) are shown separately. **(D)** The enriched KEGG pathways of the new targets of hsa-let-7a-2_4c. **(E)** The most significant GO terms of the new targets of hsa-let-7a-2_4c. The GO terms with the smallest corrected P-value in the three GO categories, i.e., Molecular Function (MF), Cellular Component (CC) and Biological Process (BP), were presented respectively.

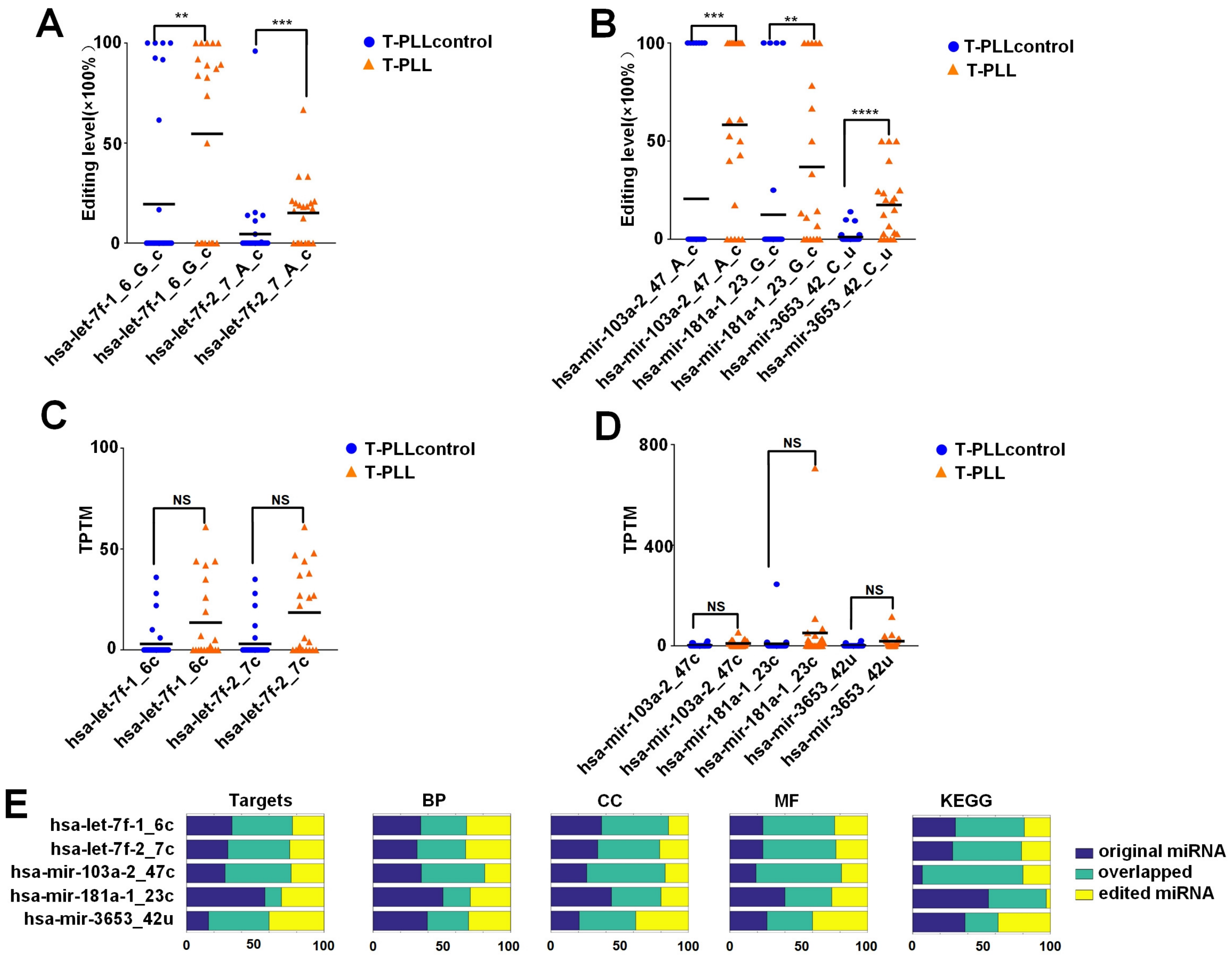

Figure S6. Target analysis of original and edited miRNAs in T-PLL. **(A)** - **(B)** The comparisons of the editing levels of four 5'- editing sites (hsa-let-7f-1_6_G_c, hsa-let-7f-2_7_A_c, hsa-mir-103a-2_47_A_c and hsa-mir-181a-1_23_G_c) and one C-to-U editing site (hsa-mir-3653_42_C_u) in T-PLL and T-PLLcontrol samples. "****", "***", and "**" indicate corrected P-value < 0.0001, 0.001 and 0.01, respectively, Mann-Whitney U-tests. **(C)** - **(D)** The comparisons of the expression levels (TPTM) of four 5'- edited miRNAs (hsa-let-7f-1_6c, hsa-let-7f-2_7c, hsa-mir-103a-2_47c, and hsa-mir-181a-1_23c) and one C-to-U edited miRNA (hsa-mir-3653_42u) in T-PLL and T-PLLcontrol samples. "NS" indicates not significant, i.e., corrected P-value > 0.05, edgeR. **(E)** The comparisons of targets, GO terms, and KEGG pathways of original and edited miRNAs. The GO terms Biological Process (BP), Cellular Component (CC) and Molecular Function (MF) were shown separately.
